# Death in the Dental Chair

**Published:** 1884-02

**Authors:** 


### EDITORIAL, ETC.

---

DEATH IN THE DENTAL CHAIR.—The following account has been sent to the JOURNAL, of a recent fatal inhalation of an anæsthetic administered for the purpose of having teeth extracted.

Mrs. James Stevenson, a lady thirty-six years old, mother of seven children, the youngest aged four months, yesterday went from her home on North Main Avenue, Providence, Pennsylvania to the dental office of Dr. W. H. Heist. The time was 2:45 P. M. She was accompanied by her physician, Dr. A. Strang, of Providence, who at three o'clock began the administration of a mixture of equal parts of chloroform and ether to produce anæsthesia preparatory to the removal of sixteen teeth, with the view of having artificial dentures inserted. Mrs. Stevenson was more than ordinarily in dread of the operation, but was anxious to undergo it, not only because she desired artificial teeth, but because she attributed the cause of a neuralgic affection to the decayed fangs in her mouth. She desired to take the anæsthetic, and Dr. Strang examined her very carefully as to the condition of her lungs and heart, and found her a proper subject for the administration of the soporific. The ether and chloroform were of the best quality that could be obtained. Dr. Strang gave the anæsthetic and Dr. Heist extracted the teeth. The physician noted the condition of the pulsation and respiration carefully during the operation. In two minutes after the first inhalation two teeth were taken out and the lady revived, rose to a sitting posture, spit out the blood, and lay back again. More of the chloroform and ether was given, and then nine teeth were removed, after which she again revived, spit, rested, and asked if the teeth were not all out. The anæsthetic was again applied and five more teeth were taken out, completing the work of extracting. About two minutes after the last tooth was taken out she threw her head vioiently back, rolled up her eyes, and apparently fainted away. All the remedies usual in cases of swooning were resorted to. Cold water was dashed in her face, artificial respiration was performed, then hypodermic injections of brandy were made without any response whatever in the way of revival. Another physician was called in, and arrived in ten minutes after she had fainted. Her pulse was then beating faintly, but no effort could restore her to animation. Mrs. Stephenson was dead.

A corner's jury rendered the following verdict :

" We, the jury, do conclude and agree in finding from the evidence of witnesses and the result of the post-mortem examination, that the said Mrs. James Stephenson came to her death on the 7th of February, 1884, in the dental office of W. H. Heist, from a fainting fit, caused by excitement and shock incident to the extraction of a number of teeth, and that there was sufficient disease of the heart—though not readily recognizable during life—to cause death by any unusual excitement."

The post-mortem revealed the lungs in a condition of collapse and somewhat congested, proving that breathing stopped before the heart ceased to pulsate. The right side of the heart was dilated and had thin, weak walls. There was no valvular disease of the heart. On the top of the heart was found a small cystic tumor, that seemed to press upon the artery—the coronal artery—that supplies the heart with nourishment. This tumor, by pressing upon an important nerve, might have been a considerable factor in causing her death. The stomach showed a catarrh condition, and gastritis was indicated.

The practice of giving chloroform or other anæsthetic in a sitting posture is so universal that the jury did not feel like expressing its condemnation of the practice, but it is none the less reprehensible. In this case the presence of the family physician, endorsing the practice, seemed to remove this matter beyond the province of the jury.

This point, was considered, however, that while the use of chloroform a short time previously may have been one factor in producing the tendency to fainting, the evidence was such as to disconnect it from playing an all-important part in the cause of death.

The public sentiment of the Providence community and the friends of the victim is favorable towards exonerating the physician and dentist from all blame. The jurors and coroner were also in warm sympathy with the famiiy, and agreed to devote their fees to the bereaved ones.

The case is a sad one and calls for unusual sympathy, not only towards the friends of the deceased, but to the very unfortunate position in which the physician and dentist are placed.

